# Angiotensin II centrally induces frequent detrusor contractility of the bladder by acting on brain angiotensin II type 1 receptors in rats

**DOI:** 10.1038/srep22213

**Published:** 2016-02-24

**Authors:** Bunya Kawamoto, Shogo Shimizu, Takahiro Shimizu, Youichirou Higashi, Masashi Honda, Takehiro Sejima, Motoaki Saito, Atsushi Takenaka

**Affiliations:** 1 Division of Urology, Department of Surgery, Tottori University Faculty of Medicine, Yonago, Japan; 2 Department of Pharmacology, Kochi Medical School, Kochi University, Nankoku, Japan

## Abstract

Angiotensin (Ang) II plays an important role in the brain as a neurotransmitter and is involved in psychological stress reactions, for example through activation of the sympatho-adrenomedullary system. We investigated the effects of centrally administered Ang II on the micturition reflex, which is potentially affected by the sympatho-adrenomedullary system, and brain Ang II receptors in urethane-anesthetized (1.0 g/kg, intraperitoneally) male rats. Central administration of Ang II (0.01, 0.02, and 0.07 nmol per rat, intracerebroventricularly, icv) but not vehicle rapidly and dose-dependently decreased the urinary bladder intercontraction interval, without altering the bladder detrusor pressure. Central administration of antagonists of Ang II type 1 but not type 2 receptors inhibited the Ang II-induced shortening of intercontraction intervals. Administration of the highest dose of Ang II (0.07 nmol per rat, icv) but not lower doses (0.01 and 0.02 nmol per rat, icv) elevated the plasma concentration of adrenaline. Bilateral adrenalectomy reduced Ang II-induced elevation in adrenaline, but had no effect on the Ang II-induced shortening of the intercontraction interval. These data suggest that central administration of Ang II increases urinary frequency by acting on brain Ang II type 1 receptors, independent of activation of the sympatho-adrenomedullary system.

Increasing evidence indicates that psychological stress exacerbates many pathophysiological conditions, such as insomnia and cardiovascular disease^1^,^2^. Additionally, psychological stress plays an important role in urinary frequency and lower urinary tract dysfunction, including overactive bladder and painful bladder syndrome/interstitial cystitis^3^,^4^. Smith *et al.* reported that water-avoidance stress increases urinary frequency and decreases the voiding interval^3^. Clinical data have shown that children with a recent life stressor or psychiatric disorder have significantly greater lower urinary tract symptom scores than healthy children^5^. Bogner *et al.* reported that urinary incontinence related to condition-specific functional loss is associated with higher rates of psychological distress^6^. Despite a body of evidence indicating that psychological stress plays an important role in voiding disorders, the pathophysiological mechanism underlying stress-induced increase in urinary frequency is not well understood.

Angiotensin II (Ang II) is a well-known peptide hormone that causes vasoconstriction, leading to an increase in blood pressure (BP)^7^. Ang II receptors are divided into the Ang II type 1 (AT1) and Ang II type 2 (AT2) subtypes, and are expressed in the brain^8^. Circulating Ang II is poorly transferred into the brain via the circumventricular organs, which are blood-brain barrier deficient, where Ang II receptors are located^9^. In the brain, Ang II acts as neuropeptide, neuromodulator, and neurotransmitter^10^.

Psychological stress increases Ang II as a stress hormone in order to modulate the neuroendocrine system and influence behaviour^11^. Repeated immobilization stress produces a significant increase in the density of Ang II binding sites in the rat paraventricular nucleus and subfornical organ^12^.

Activation of the sympathetic nervous system by stressors increases the release of catecholamines, such as noradrenaline (NA) and adrenaline (Ad)^13^. Plasma NA is released from sympathetic nerve terminals and is secreted from NA-containing cells in the adrenal medulla, whilst plasma Ad is mainly secreted from Ad-containing cells in the adrenal medulla^14^,^15^. A previous report demonstrated that intraperitoneally administered NA and alpha 1 or alpha 2 adrenoceptor agonists increases the frequency of voiding contraction in a dose-dependent manner^16^. Thus, it is also possible that the sympatho-adrenomedullary (SA) system affects the micturition reflex.

We have previously shown that central Ang II is involved in activation of the SA system via brain AT1 receptors (AT1R)^7^. The aim of the current study was to investigate the relationship between central Ang II as a neurotransmitter and the micturition reflex.

## Results

### Central Ang II actions in micturition reflex

**Figure 1:**
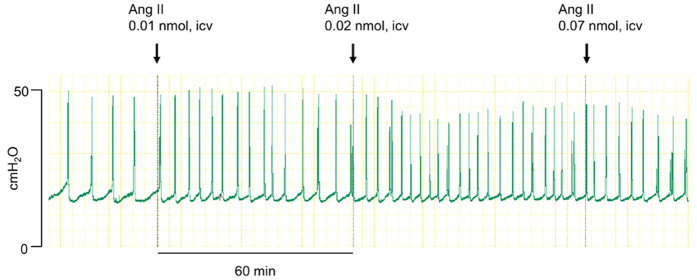
Representative urodynamic recording of the effect of centrally administered Ang II in the rat.

Centrally administered vehicle (1, 2, and 7 μl, intracerebroventricularly [icv]) had no effect on the urinary bladder intercontraction interval (ICI). Alternatively, centrally administered Ang II at the lowest dose (0.01 nmol per rat, icv) rapidly and significantly decreased the ICI as compared to baseline (Table 1). This response was sustained for at least 1 h after the first administration (Fig. 1). Thereafter, serially administered Ang II into the brain at higher doses (0.02 and 0.07 nmol, icv) rapidly and significantly shortened the ICI as compared to baseline. In contrast, centrally administered vehicle or Ang II had no effect on the maximum voiding pressure (MVP).

A centrally administered AT1R-selective antagonist, valsartan (10 nmol per rat, icv), significantly diminished the Ang II (0.01 and 0.02 nmol per rat, icv)-induced decrease in ICI. Pretreatment with a centrally administered AT2R-selective antagonist, PD123319, or peripherally administered valsartan or PD123319 (intravenous, iv) had no effect on the Ang II-induced decrease in ICI.

Moreover, acute bilateral adrenalectomy (ADX) did not alter the central Ang II-induced decrease in ICI (Table 1). Of note, in our preliminary data, there was no significant difference in the post-micturition residual urine volume between the icv vehicle-treated group and the icv Ang II-treated group (data not shown).

### Effect of centrally administered Ang II on plasma NA and Ad

**Figure 2:**
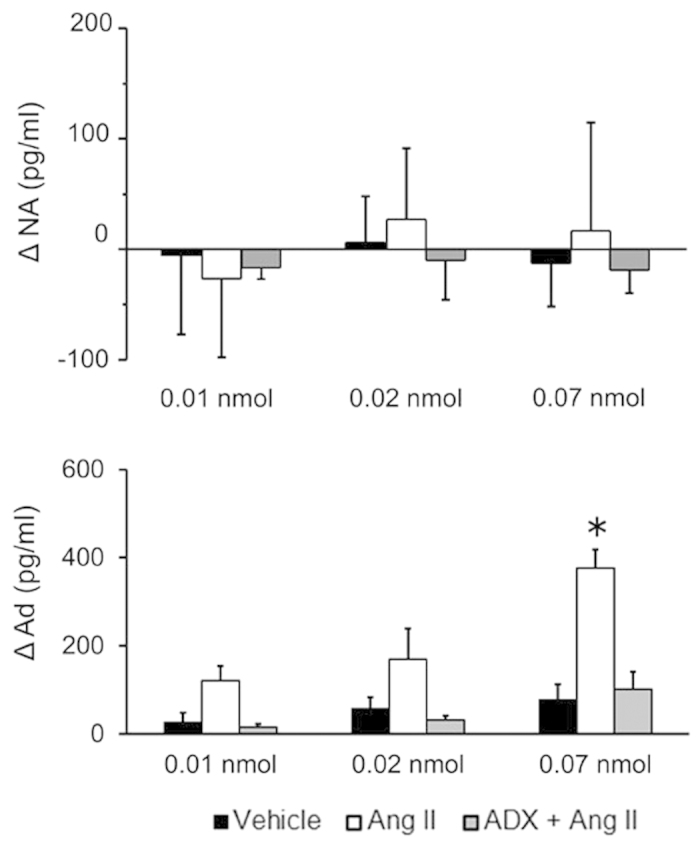
Plasma noradrenaline and adrenaline concentrations. NA: noradrenaline; Ad: adrenaline; ΔNA and ΔAd: increments of NA and Ad measured 5 min after each central Ang II administration, in comparison with NA and Ad measured 5 min before the initial central Ang II administration. Values are reported as means ± SEM. Vehicle: Wistar rats icv administered vehicle; Ang II: Wistar rats icv administered Ang II; ADX + Ang II: adrenalectomised Wistar rats icv administered Ang II. *P < 0.05 using the Turkey-Kramer’s test to compare with the Vehicle group or the ADX + Ang II group, respectively.

Peripheral plasma catecholamine (NA and Ad) levels were measured to evaluate the activation of the SA system induced by centrally administered Ang II (0.01, 0.02 and 0.07 nmol per rat, icv). There was no significant difference in the concentration of plasma NA between the icv vehicle-treated control group and the icv Ang II-treated group at 5 min post-administration (Fig. 2). Alternatively, centrally administered Ang II at the highest dose (0.07 nmol per rat, icv) but not at lower doses (0.01 and 0.02 nmol per rat, icv) significantly elevated the concentration of plasma Ad as compared to the volume-matched vehicle control. ADX significantly inhibited the Ang II-induced elevation of plasma Ad. Moreover, there were no significant differences in the plasma concentrations of NA or Ad between the icv vehicle-treated group and the icv Ang II-treated group after ADX (Fig. 2).

### Effect of centrally administered Ang II on BP

**Figure 3:**
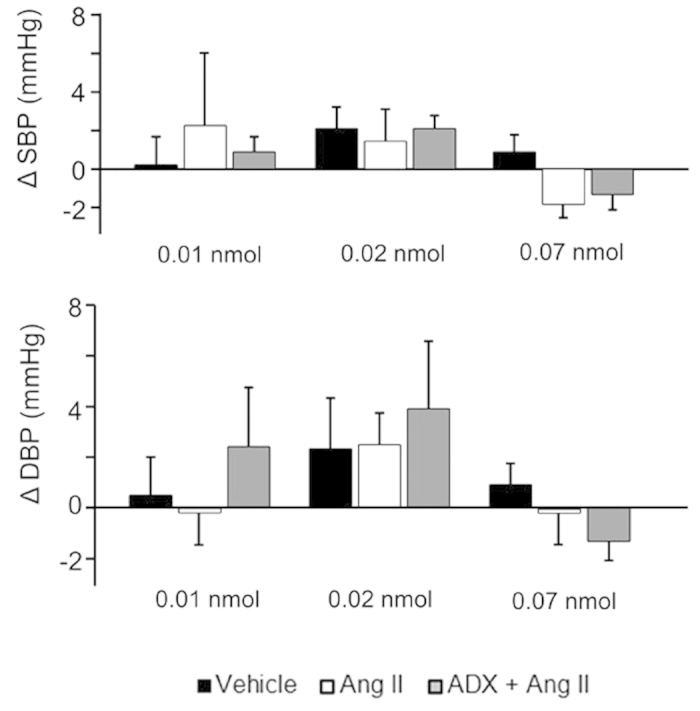
Effects of centrally administered Ang II on blood pressures. ADX: acute bilateral adrenalectomy; SBP: systolic blood pressure; DBP: diastolic blood pressure. ΔSBP and ΔDBP: changes in SBP and DBP 5 min after each central Ang II administration, in comparison to SBP and DBP measured 5 min before the initial administration. Values are reported as means ± SEM. Vehicle: Wistar rats icv administered vehicle; Ang II: Wistar rats icv administered Ang II; ADX + Ang II: adrenalectomised Wistar rats icv administered Ang II.

Centrally administered Ang II (0.01, 0.02, and 0.07 nmol per rat, icv) had no significant effect on changes in systolic and diastolic blood pressure (SBP and DBP) as compared to the volume-matched vehicle control. There was also no significant difference in changes in SBP and DBP between the Ang II group and Ang II-pretreated ADX group (Fig. 3).

## Discussion

The present study shows that central administration of Ang II dose-dependently shortens the ICI, without affecting MVP in rats. Pretreatment with peripheral administration of valsartan, an AT1R-selective antagonist, or PD123319, an AT2R-selective antagonist, failed to inhibit the Ang II-induced decrease in ICI. Alternatively, central administration of valsartan, but not PD123319, prevented the Ang II-induced decrease in ICI. Administration of Ang II at the highest dose (0.07 nmol per rat, icv) but not at lower doses (0.01 and 0.02 nmol per rat, icv) elevated the plasma concentration of Ad, but not plasma NA. ADX, which was performed to eliminate the effects of plasma Ad and NA, had no effect on the Ang II-induced decrease in ICI. Central administration of Ang II had no significant effects on changes in SBP and DBP as compared to the volume-matched vehicle control. These data indicate that brain Ang II and AT1Rs are involved in the facilitation of the micturition reflex in the rat, independent of modulation of the SA system. To our knowledge, this is the first study to show that centrally administered Ang II dose-dependently shortens the ICI without affecting MVP.

There is some evidence indicating an influence of the renin-angiotensin system on the activity of the lower urinary tract. Ito and colleagues reported that administration of AT1R antagonists significantly reduced the International Prostate Symptom Score in male hypertension patients. Moreover, the frequency score was significantly improved in the group receiving combined medications that included AT1R antagonists^17^. Previous preclinical research demonstrated that peripheral Ang II causes urethral contractions in a dose-dependent manner, and that chronic treatment with an AT1R antagonist, losartan, reduces urinary frequency in ovariectomised oestrogen-deficient rats^18^. Furthermore, Comiter *et al.* reported that losartan partially prevents urodynamic and structural changes associated with bladder obstruction in the mouse^19^. These previous studies suggest that peripheral administration of AT1R antagonists is useful for reducing urinary frequency. However, the central effect of Ang II on the lower urinary tract is not well understood.

Stimulation of parasympathetic nerves causes the release of acetylcholine and muscarinic effects that result in the contraction of the detrusor muscle and relaxation of the trigone^20^. Subsequently, a rise in intravesical pressure results in micturition. However, our study showed that centrally administered Ang II failed to alter the MVP. These data indicate that central Ang II-induced bladder stimulation may not be attributed to activation of parasympathetic nerves. Moreover, in our preliminary data, there was no significant difference in the post-micturition residual urine volume between the vehicle-treated group and the Ang II-treated group (data not shown). Merrill *et al.* similarly reported that repeated variate stress decreases the ICI without causing changes in bladder pressure or residual urine volume in conscious rats^4^. Therefore, the central Ang II-induced urodynamic phenotype may be similar to that induced by repeated variate stress.

Physiological and/or psychological stress increases circulating plasma renin and Ang II^11^,^13^. In a previous report, we showed that centrally administered Ang II (3 nmol per rat, icv) induces the secretion of Ad but not NA from the adrenal medulla in the rat, indicating activation of the SA system^7^. However, our current study shows that centrally administered Ang II causes weak activation of the SA system at low doses (0.01 and 0.02 nmol per rat, icv). Moreover, centrally administered Ang II at the highest dose (0.07 nmol per rat, icv) induced the elevation of plasma Ad, and this elevation was almost completely abolished by ADX, in agreement with our previous data^7^. Of note, ADX had no effect on shortening of the ICI induced by Ang II at the highest dose (0.07 nmol per rat, icv). Thus, centrally administered Ang II induced the micturition reflex independently of Ang II activation of the SA system. Although peripheral Ang II is known to be a strong vasopressor, brain Ang II and AT1Rs have also been implicated in the regulation of BP^7^. Our previous reports showed that centrally administered Ang II (3 nmol per rat, icv) elevated SBP and DBP, and that these increases were abolished by valsartan in the rat^7^. However, the current study showed that centrally administered Ang II (0.01, 0.02, and 0.07 nmol per rat, icv) failed to elevate SBP and DBP in the rat. This suggests that central administration of Ang II induces the shortening of the ICI without affecting BP.

In the central nervous system, AT1Rs are distributed widely throughout the brain^21^. AT1Rs are richly expressed in the cortex, hippocampus, locus coeruleus (LC), hypothalamic paraventricular nucleus (PVN), and nucleus tractus solitaries, which are brain regions responsible for the response to stressors^10^,^12^. Moreover, AT1Rs have been detected in the periaqueductal grey (PAG) area^22^. The LC is involved in controlling the physiological response to stress and panic^10^,^12^. The PVN, which is in the hypothalamus and located adjacent to the third ventricle of the forebrain, is another important brain region for stress reaction^23^. The PAG receives many afferent inputs from the spinal cord and descending neurons project from the PAG to the rostral ventrolateral medulla to regulate autonomic activity^24^. The PAG also regulates Barrington’s nucleus, which controls the neurons in the pontine micturition centre^25^. A previous report showed that the PVN directly influences the PAG through these nervous fibres^26^. Yang *et al.* reported that stimulation of the PVN increases the secretion of arginine vasopressin, which could influence the activity of the PAG^27^. Psychological stress increases the release of Ang II and AT1R expression in the PVN^28^. Xing *et al.* demonstrated that Ang II inhibits gamma-aminobutyric acid synaptic inputs to the dorsolateral region of the PAG through activation of presynaptic AT1Rs^24^. These data suggest that centrally administered Ang II could affect the micturition reflex through exogenous activation of the projection from the PAG to Barrington’s nucleus. Further studies are required to elucidate the mechanism underlying the effect of central Ang II and AT1Rs on the micturition reflex.

Although most AT1R antagonists including valsartan do not penetrate the blood-brain barrier sufficiently^8^, some peripherally administered AT1R antagonists distribute both outside and inside of the blood-brain barrier^29^. Braszko *et al.* suggested that oral administration of candesartan has a memory-enhancing effect and improves inhibitory avoidance performance in rats^30^. Oral administration of telmisartan effectively restores cognitive functions impaired by psychological stress^31^. These data suggest that AT1R antagonists have utility for stress-induced or psychological urinary frequency.

The main limitation of the current study is that only acute response to central Ang II and AT1R antagonists were investigated. Additionally, previous studies have shown that neuronal as well as peripheral AT1Rs play a role in sodium reabsorption, BP maintenance, and vasopressin release^32^,^33^. Therefore, central AT1R antagonists could inhibit sodium reabsorption and increase urine volume; however, in our study, we observed no acute effect of central Ang II on urine volume. The effects of chronic central administration of Ang II or AT1R antagonists on urine volume and micturition require further examination.

## Conclusions

Centrally administered Ang II acting via brain AT1Rs induces the micturition reflex. This finding suggests that AT1R antagonists may be useful for reducing urinary frequency.

## Methods

### Animals

All experiments were approved by Kochi University (No. H-68) and were conducted in accordance with the guidelines for the care and use of laboratory animals, which conformed to the “Guidelines for Proper Conduct of Animal Experiments” developed by the Science Council of Japan. All studies involving animals are reported in accordance with the “Animals in Research: Reporting In Vivo Experiments” guidelines^34^. All efforts were made to minimize animal suffering and the number of animals needed to obtain reliable results.

Male Wistar rats (Japan SLC Inc., Hamamatsu, Japan), weighing 360–410 g were used in this study. The rats were purchased when they were 8 weeks old, and they were kept under identical temperature and humidity conditions *ad libitum* with access to food (laboratory chow CE-2; Clea Japan, Hamamatsu, Japan) and drinking water.

### Experimental procedures

In the morning (9:00–10:00), rats were anesthetized with urethane (1.0 g/kg, intraperitoneally) (Sigma-Aldrich, St Louis, MO, USA). Rats were then cannulated at the femoral vein for saline infusions (1.2 ml/h) and for the iv administration of drugs, and were cannulated at the femoral artery using PE-50 tubing connected to a pressure transducer for measurement of BP and collection of blood samples^35^. The bladder dome was cannulated with a PE-50 catheter, which was connected to a pressure transducer (DX-100; Nihon Koden, Tokyo, Japan) and a pump for saline infusions (12 ml/h) (5200; TOP, Tokyo, Japan). The signal provided by the transducer was monitored by a personal computer (Macintosh G4; Apple Inc., Cupertino, CA, USA) that was connected via a bridge amplifier (ML112; AD-Instruments, Pty Ltd, Castle Hill, Australia) and a multiport controller (PowerLab/8sp; AD-Instruments, Pty Ltd) for measuring the BP and intravesical pressure data. In some experiments, ADX (plus hydrocortisone, 5 mg/kg per rat, intramuscularly) was performed before cannulation by an abdominal midline incision^7^,^35^. ADX was completed 30 min before stereotaxic fixation for icv administration. Rats underwent stereotaxis with an SR-6R apparatus (Narishige, Tokyo, Japan) and remained fixed until the end of the experiment. The skull was drilled and a burr hole opening was made above the right ventricle, which was 0.8 mm posterior, 1.5 mm right from bregma, and 4.0 mm below the surface of the brain, with the rat in a prone position as previously described^36^. Rats were stabilized thereafter for at least 3 h before the administration of drugs.

### Drug administration

**Figure 4:**
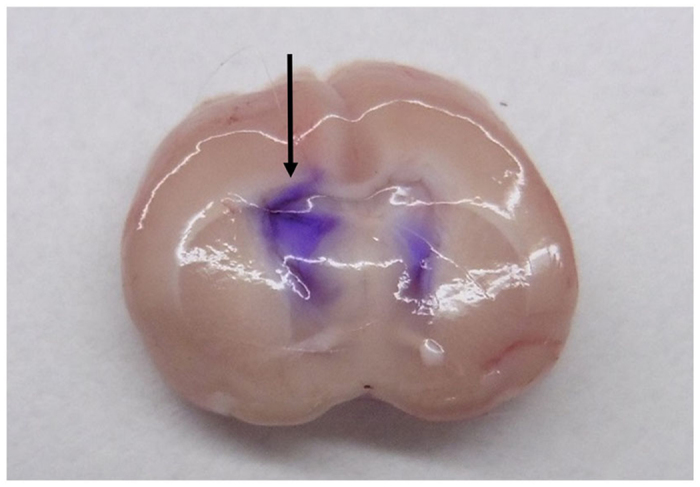
Experimental rat brain section. Crystal violet was centrally administered at the conclusion of all experiments to confirm the accuracy of the icv injection site. The arrow shows the injected line and purple stain indicates the spread of the crystal violet dye.

Ang II was dissolved in sterile deionized water and slowly administered into the right ventricle during the stereotaxic procedure using a cannula connected to a 10-μl Hamilton syringe, and the cannula remained in place until the end of the experiment^7^. Valsartan, a selective AT1R antagonist, and PD123319, a selective AT2R antagonist, were slowly administered icv using the cannula-connected 10-μl Hamilton syringe, which was kept in the ventricle for 15 min to prevent leakage of the drug. Antagonists were icv or iv administered 30 min before Ang II administration. Ang II solution (0.01 nmol/μl) was icv administered at 3 doses: 0.01 nmol (1 μl), 0.02 nmol (2 μl), and 0.07 nmol (7 μl) at an interval of 1 h. Cystometry was performed every hour after each icv administration. The accurate location of the injected cannula in the brain was confirmed at the end of each experiment by crystal violet injection through the cannula, which was observed to spread throughout the entire ventricular system^7^ (Fig. 4).

### Experimental group

Rats were categorized into 7 groups, as follows: 1) vehicle control that was administered sterile deionized water icv (n = 7); 2) icv-administered Ang II (0.01, 0.02, and 0.07 nmol per rat; n = 7); 3) 10 nmol valsartan (3 μl) icv-administered before icv-administration of Ang II (n = 5); 4) 100 nmol PD123319 (5 μl) icv-administered before icv-administration of Ang II (n = 5); 5) 100 nmol valsartan (200 μl) iv-administered before icv-administration of Ang II (n = 5); 6) 100 nmol PD123319 (200 μl) iv-administered before icv-administration of Ang II (n = 5); and 7) ADX with hydrocortisone (5 mg/kg per rat, intramuscular injection) administered before icv-administration of Ang II (n = 4). Central administration of 10 nmol valsartan was performed because the icv-administration of 100 nmol valsartan caused hypotensive effects in our preliminary study. Moreover, centrally administered valsartan (10 nmol) or PD123319 (100 nmol) was chosen because these drugs failed to affect urodynamic parameters in rats.

### Cystometry

After stabilization for 3 h, cystometry was performed whilst the rats were in a prone position^35^. The values of urodynamic parameters were calculated from the data obtained 10 min after Ang II administration because Ang II is metabolized rapidly and has a half-life of <2 min at 37 °C^37^. Relative values of ICI and MVP were calculated as the ratio of the average of those values measured for 10 min after the administration of the drug to the average of those values measured 10 min before the initial Ang II administration. MVP was calculated as the average of those values measured for 10 min after administration.

### Measurement of plasma NA and Ad

Blood samples (400 μl) were collected from the arterial catheter before initial icv-administration of Ang II and 5 min after each icv-administration of Ang II in all rats. Plasma was prepared immediately after the animal experiments. Plasma NA and Ad were extracted using the method described by Anton and Sayre with slight modifications^35^,^38^, and were then assayed using high-performance liquid chromatography as previously described^7^.

### Statistical analyses

All of the values are expressed as the means ± the standard errors of the means (SEM). Statistical differences were determined using a one-way analysis of variance (ANOVA), followed by post hoc analysis. In Table 1, 3 multiple comparisons were made versus the baseline in each group using the Bonferroni correction. In Figs 2 and 3, all possible comparisons were made using the Turkey-Kramer’s test. P < 0.05 was considered to be statistically significant.

### Drugs and chemicals

The following drugs were used: Ang II (Peptide Institute, Osaka, Japan); valsartan [(*S*)-3-methyl-2-[*N*-({4-[2-(2*H*-1,2,3,4-tetrazol-5-yl)phenyl]phenyl}methyl)pentanamido]butanoic acid] (Cayman Chemical, Ann Arbor, MI, USA); PD123319 (PD123319 ditrifluoroacetate) [(*S*)-1-[[4-(dimethylamino)-3-methylphenyl]methyl]-5-(diphenylacetyl)-4,5,6,7-tetrahydro-1*H*-imidazo[4,5-c]pyridine-6-carboxylic acid ditrifluoroacetate] (R&D Systems, Inc., Minneapolis, MN, USA). All other chemicals were obtained commercially and were reagent grade.

## Additional Information

**How to cite this article**: Kawamoto, B. *et al.* Angiotensin II centrally induces frequent detrusor contractility of the bladder by acting on brain angiotensin II type 1 receptors in rats. *Sci. Rep.* **6**, 22213; doi: 10.1038/srep22213 (2016).

## Figures and Tables

**Table 1:** Urodynamic parameters in experimental rats.

	Vehicle	Ang II	Val icv + Ang II	PD icv + Ang II	Val iv + Ang II	PD iv + Ang II	ADX + Ang II
ICI (% of basal level)							
Basal level	100 ± 0	100 ± 0	100 ± 0	100 ± 0	100 ± 0	100 ± 0	100 ± 0
0.01 nmol	104 ± 5	76 ± 6*	97 ± 4	76 ± 10*	60 ± 4*	72 ± 7*	76 ± 7*
0.02 nmol	96 ± 5	66 ± 5*	91 ± 7	68 ± 5*	58 ± 5*	63 ± 11*	67 ± 9*
0.07 nmol	98 ± 5	49 ± 5*	80 ± 5*	55 ± 7*	57 ± 9*	65 ± 11*	57 ± 7*
MVP (% of basal level)							
Basal level	100 ± 0	100 ± 0	100 ± 0	100 ± 0	100 ± 0	100 ± 0	100 ± 0
0.01 nmol	98 ± 2	98 ± 4	98 ± 3	105 ± 4	93 ± 5	102 ± 5	107 ± 10
0.02 nmol	99 ± 3	105 ± 4	106 ± 2	106 ± 3	101 ± 4	108 ± 8	114 ± 11
0.07 nmol	99 ± 4	98 ± 4	106 ± 2	107 ± 3	103 ± 5	106 ± 7	115 ± 12

Ang II solution (0.01 nmol/μl) was intracerebroventricularly (icv) administered at 3 doses: 0.01 nmol (1 μl), 0.02 nmol (2 μl), and 0.07 nmol (7 μl), at an interval of 1 h.

Cystometry was performed every hour after each icv administration. The ICI and MVP values in each rat before the first Ang II administration were set as 100%. ICI: intercontraction interval; MVP: maximum voiding pressure; ADX: acute bilateral adrenalectomy; Vehicle: Wistar rats icv administered vehicle (1, 2, and 7 μl); Ang II: Wistar rats icv administered Ang II solution (0.01 nmol/μl) at 3 doses: 0.01 nmol (1 μl), 0.02 nmol (2 μl), and 0.07 nmol (7 μl), at an interval of 1 h; Val icv + Ang II: Wistar rats icv administered valsartan at 10 nmol before icv administration of Ang II; PD icv + Ang II: Wistar rats icv administered PD123319 at 100 nmol per rat before icv administration of Ang II; Val iv + Ang II: Wistar rats iv administered valsartan at 100 nmol before icv administration of Ang II; PD iv + Ang II: Wistar rats iv administered PD123319 at 100 nmol per rat before icv administration of Ang II; ADX + Ang II: adrenalectomised Wistar rats icv administered Ang II. Values are reported as means ± SEM.

*P < 0.05, as compared with the respective parameters before administration of Ang II using the Bonferroni correction.
